# Neutralization of the autophagy-repressive tissue hormone DBI/ACBP (diazepam binding inhibitor, acyl-CoA binding protein) enhances anticancer immunosurveillance

**DOI:** 10.1080/15548627.2024.2411854

**Published:** 2024-10-17

**Authors:** Léa Montégut, Isabelle Martins, Guido Kroemer

**Affiliations:** a Centre de Recherche des Cordeliers, Equipe labellisée par la Ligue contre le cancer, Inserm U1138, Université Paris Cité, Sorbonne Université, Paris, France; b Metabolomics and Cell Biology Platforms, Gustave Roussy Institut, Villejuif, France; c Department of Biology, Institut du Cancer Paris CARPEM, Hôpital Européen Georges Pompidou, AP-HP, Paris, France

**Keywords:** Autophagy, cancer, immunotherapy, metabolism, obesity

## Abstract

The plasma concentration of the macroautophagy/autophagy inhibitor DBI/ACBP (diazepam binding inhibitor, acyl-CoA binding protein) increases with aging and body mass index (BMI). Both advanced age and obesity are among the most important risk factors for the development of cancer. We observed that patients with cancer predisposition syndromes due to mutations in *BRCA1*, *BRCA2* and *TP53* exhibit abnormally high plasma DBI/ACBP levels. Additionally, patients without known cancer predisposition syndromes also manifest higher DBI/ACBP levels before imminent cancer diagnosis (within 0–3 years) as compared to age and BMI-matched controls who remain cancer-free. Thus, supranormal plasma DBI/ACBP constitutes a risk factor for later cancer development. Mouse experimentation revealed that genetic or antibody-mediated DBI/ACBP inhibition can delay the development or progression of cancers. In the context of chemoimmunotherapy, DBI/ACBP neutralization enhances tumor infiltration by non-exhausted effector T cells but reduces infiltration by regulatory T cells. This resulted in better cancer control in models of breast cancer, non-small cell lung cancer and sarcoma. We conclude that DBI/ACBP constitutes an actionable autophagy checkpoint for improving cancer immunosurveillance. **Abbreviation**: BMI, body mass index; CTL, cytotoxic T lymphocyte; DBI, diazepam binding inhibitor, acyl-CoA binding protein; mAb, monoclonal antibody; NSCLC, non-small cell lung cancer; PDCD1/PD-1, programmed cell death 1; scRNA-seq, single-cell RNA sequencing; T_reg_, regulatory T cell.

DBI/ACBP (diazepam binding inhibitor, acyl-CoA binding protein) functions as an extracellular autophagy checkpoint. Intriguingly, this protein appears to mediate pro-aging effects, commensurate with the established anti-aging effects of autophagy. Thus, knockout of the genes encoding orthologs of DBI/ACBP in yeast and nematodes causes lifespan extension. In human populations, plasma concentrations of DBI/ACBP increase with age and body mass index (BMI), and both these correlations are statistically independent from each other. Additionally, in a population of healthy volunteers enrolled in a longitudinal follow-up of disease development, elevated plasma levels of DBI/ACBP are observed prior to cardiovascular events. In a similar approach, we examined whether measurements of plasma DBI/ACBP would make it possible to predict the development of cancer, another age-related disease, in still (apparently) healthy individuals [1]. We found that neoplastic disease is preceded by a surge in plasma DBI/ACBP that is significant compared to individuals that are matched for major epidemiological characteristic (such as sex, age, BMI and known risk factors including tobacco abuse) but do not develop cancer (Figure 1A). This observation suggests that DBI/ACBP is not only a biomarker of chronological aging – because it increases with age measured in years – but also a biomarker of biological aging – because its elevation precedes the development of age-related diseases.

**Figure 1.**
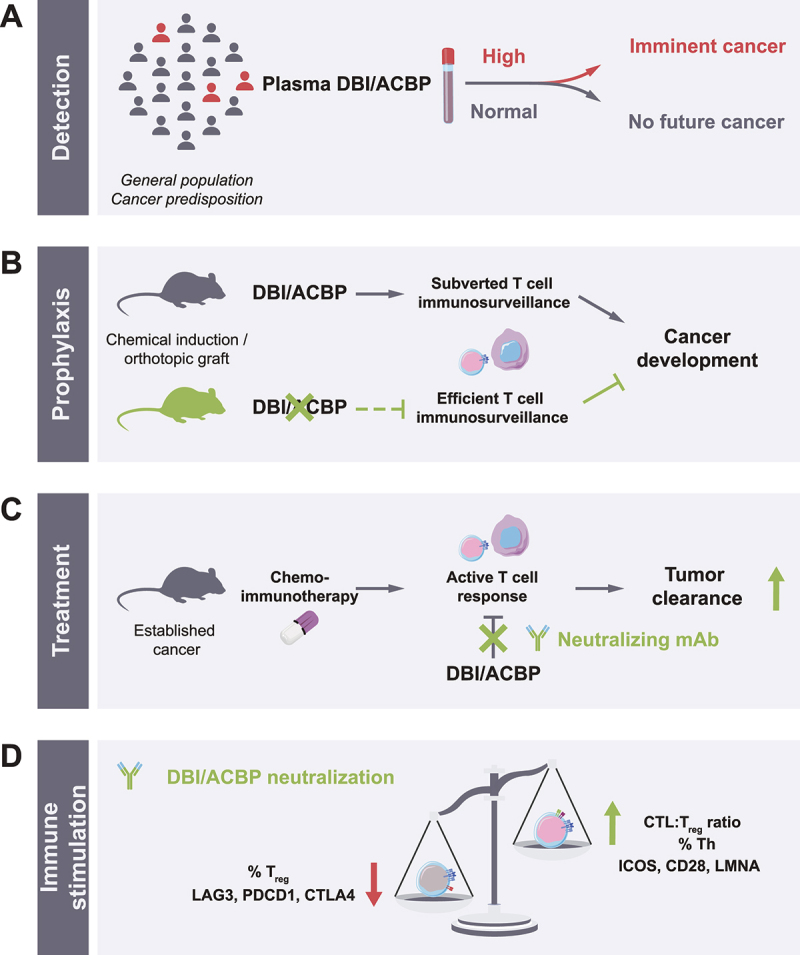
Evidence in favor of the procarcinogenic and cancer immunosurveillance-suppressive action of DBI/ACBP. (A) Imminent cancer diagnosis in patients with high DBI/ACBP levels. (B) Delayed cancer development in mice in which DBI/ACBP is inhibited. (C) Improved outcome of chemoimmunotherapy in the context of DBI/ACBP neutralization. (D) Signs of local immune activation in tumors from mice treated with chemoimmunotherapy combined with anti-dbi/acbp antibody. Icons by servier (https://smart.servier.com/), licensed under CC-BY 3.0 unported, were used and modified to generate the figure.

Knowing that DBI/ACBP neutralization can prevent anthracycline-induced cardiac aging, we decided to explore the potential pathophysiological role of DBI/ACBP in carcinogenesis, a process that is notoriously age-related. For this we first compared mammalian carcinogenesis in mice that lacked DBI/ACBP due to the inducible knockout of the *Dbi* gene and in control mice. Mammalian carcinogenesis induced by a combination of synthetic progesterone and DNA damage is affected by the *dbi* knockout in a peculiar fashion. While the appearance of tumors is not influenced by DBI/ACBP, the *dbi* knockout did slow down the growth of such tumors. Because the neoformation of mammary carcinomas is under immunosurveillance by NK (but not T) cells, while tumor progression of established cancers is under immunosurveillance by T (but not NK) cells, we concluded that DBI/ACBP might affect T lymphocyte-dependent immunosurveillance. Accordingly, the growth of a transplantable mammary carcinoma cell line could be retarded by DBI/ACBP inhibition using a monoclonal antibody (mAb), but only if this mAb is combined with another one targeting PDCD1/PD-1 (programmed cell death 1), which is a T cell-relevant immune checkpoint. We also found that urethane-induced lung carcinogenesis (which is under T cell-dependent control) is retarded when neutralizing autoantibodies against DBI/ACBP are induced. The orthotopic growth of a non-small cell lung cancer (NSCLC) is also reduced when DBI/ACBP is inhibited, and this effect is lost in athymic mice lacking thymus-derived T cells. Altogether, these results suggest that DBI/ACBP neutralization stimulates T cell-dependent cancer immunosurveillance (Figure 1B).

Driven by this conclusion, we wondered whether anti-DBI/ACBP mAb might be useful in the therapeutic setting, i.e., in the treatment of established cancers. For this, we combined the anti-DBI/ACBP mAb with chemoimmunotherapy (which is the current standard of care for many cancers, i.e., chemotherapy together with immunotherapy targeting PDCD1) against two types of cancers, namely NSCLC and cutaneous sarcoma. Orthotopic NSCLCs were generated by intravenous injection of luciferase-expressing TC-1 cells in immunocompetent C57BL/6 mice and then monitored by bioluminescence imaging of the thorax. Orthotopic sarcomas were induced by subcutaneous injection of MCA205 cells and then monitored by palpation and caliper-based measurements in the same mouse strain. Mice bearing either of the two tumor types similarly responded to chemoimmunotherapy, and this response was further improved by combination with the anti-DBI/ACBP mAb (Figure 1C). Hence, it appears that DBI/ACBP neutralization enhances the therapeutic efficacy of chemoimmunotherapy.

In a final twist, we decided to explore the mechanisms through which anti-DBI/ACBP mAb improves therapeutic outcome. For this we compared the phenotype of MCA205 fibrosarcoma-infiltrating T lymphocytes in four different groups of mice, namely, (i) animals receiving control treatments only (i.e., vehicle and isotype control antibodies), (ii) those receiving chemoimmunotherapy only (i.e., oxaliplatin plus anti-PDCD1 antibody), (iii) mice receiving anti-DBI/ACBP only, and (iv) tumor bearers treated with the combination of chemoimmunotherapy and anti-DBI/ACBP. We then characterized the T cell infiltrate of the tumors using two complementary methods, namely, (i) multi-color immunofluorescence phenotyping by high-dimensional cytometry, and (ii) single-cell transcriptomics by means of RNA sequencing (scRNA-seq). Both methods convergently led to similar conclusions. The combination treatment (chemoimmunotherapy + DBI/ACBP blockade) is the most efficient in increasing infiltration by cytotoxic T lymphocytes (CTL) and T helper cells, but decreasing regulatory T cells (T_reg_), hence improving the local CTL:Treg ratio (Figure 1D). Moreover, DBI/ACBP blockade increases the chemoimmunotherapy-driven surge of activated T helper cells but reduces the surge in exhausted CD8^+^ T cells, as defined by combinations of T cell exhaustion markers. At the transcriptomic level, DBI/ACBP neutralization reduces the abundance of RNA coding for CTL effector molecules (e.g., GZMB [granzyme B]), a decrease in inhibitory immune checkpoint molecules (PDCD1 and CTLA4 [cytotoxic T-lymphocyte associated protein 4) and an increase in stimulatory immune checkpoint markers (ICOS, CD28) on a per-cell basis across several T cell subpopulations. Most interestingly, all T lymphocyte subpopulations detectable by scRNA-seq exhibit an anti-DBI/ACBP mAb-induced upregulation of *Lmna*, which encodes lamin A/C, an obligatory T cell activation marker that is also endowed with potent antiaging effects.

In conclusion, four key arguments plead in favor of a major contribution of the age-associated, autophagy inhibitor DBI/ACBP to carcinogenesis and the failure of cancer immunosurveillance. First, in patients, elevations in DBI/ACBP plasma concentrations precede imminent cancer diagnosis. Second, in mice, inhibition of DBI/ACBP delays cancer development induced by carcinogens or orthotopic injection of malignant cells. Third, DBI/ACBP neutralization ameliorates the outcome of cancer treatments with chemotherapy and immunotherapy targeting PDCD1. Fourth, anti-DBI/ACBP mAb favorably influences the composition and functional state of tumor-infiltrating T lymphocytes. Thus, anti-DBI/ACBP mAb joins an expanding list of autophagy inducers (including the small molecules aspirin, 3,4-dimethoxychalcone, spermidine and thiostrepton) that mediate T cell-dependent anticancer effects.
